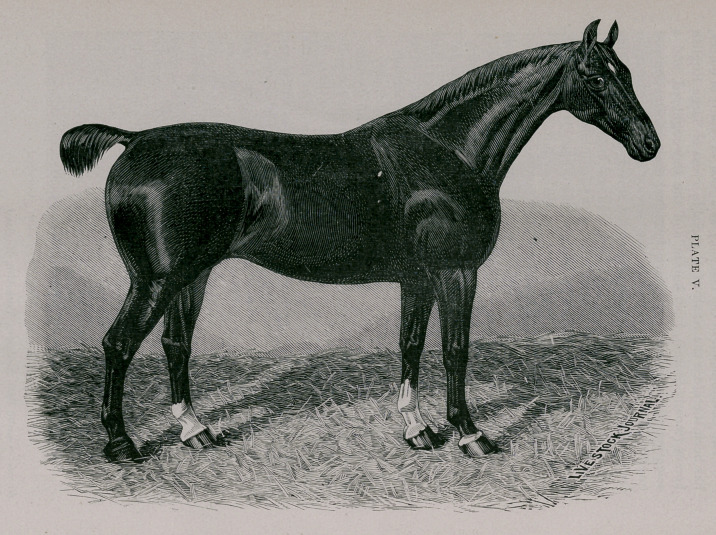# The Hackney Horse

**Published:** 1893-11

**Authors:** R. S. Huidekoper

**Affiliations:** Veterinarian; 155 West 56th Street, New York


### THE JOURNAL

OF

### COMPARATIVE MEDICINE AND VETERINARY ARCHIVES.

VOL. XIV. NOVEMBER, 1893. NO. 5.

### THE HACKNEY HORSE.

BY R. S. HUIDEKOPER, M. D., Veterinarian.*

Genus *Equus.*

Species, *Caballus.*

Race, *Hibernicus.*

Sub-division or family, *Aryano-Britannico-Hibernicus.*

When any discovery or any resuscitation of an old idea is brought before a community, it awakens at once, according to its importance, a spirit of enthusiasm or of antagonism in the minds of the people, following their individual perceptions, or from that combative disposition which lies dormant in all mankind. The pessimists accept ideas from what they hear and see with doubt, and unwilling to be convinced of anything which originates outside of themselves, except by overwhelming proof, rarely develop any of their own which are not founded on prejudice. The ultra-radical believes every thing new that he sees and hears, and in his optimism magnifies and finds chimerical futures for all he becomes interested in or undertakes. Between these two, comes the conservative worker, who is willing to weigh and investigate a new idea, test it if it offers a fair chance of return, and invest in and support it if its prospects improve after trial. The Hackney horse upon its recent introduction in numbers, into this country, was a new idea to the majority of Americans. A few gentlemen interested in sport and horseflesh commenced the importation of the Hackney, and, as they happened to be men of wealth and of prominent business or social position, they rapidly found an extremist

* The plates for illustration are kindly loaned by The American Hackney Horse Society and by George Green, Esq.

PLATE I.

ignorant following of would-be horsemen who immediately vaunted the pride of Norfolk as the only perfection of a pleasure horse, and an equally and less excusably ignorant opposition from men more experienced in the stable, who could find no merit in animals which had neither the draught weight of a steer or Clydesdale, nor the shoat-like conformation of the county fair trotter, which they had been accustomed to see trying to lower a 2.40 record.

The discussion of the Hackney's merits and faults has been so acrimoniously handled in many of the articles which have appeared in print, and it has partaken so much more of personality than of serious study, that a plain statement of facts in regard to the origin and development of this family of the horse will lead to a fairer consideration of it, and should prove of interest to all true lovers of the genus equus and to those occupied with the horse industry. The recent publication of Vol. I, "The American Hackney Stud Book," offers a reason for an additional article upon this subject at the present time, and furnishes much material of interest. Abstracts, however, will be made from the Stud Book of the English Hackney Society, as the latter's quotations from the authorities are more full and complete. Indeed, the American Book has a tendency to alteration of the meaning of the original articles, in a view to identify the Hackney as the origin of the American trotter.

Further comments will be based upon accepted facts, although the reader will be asked to await another article in the near future for the substantiation of the prehistoric and paleontological proofs of the three distinctive type characteristics of race which are found in the animal in question. The opponents of the Hackney have characterized it as a new breed, almost of mushroom or spontaneous generation, and have caricatured it by picturing a

---

PLATE I.--Irish type.

7 A.H.S.B. FASHION. 199 E.H.S.B. Imp. 1884.

*Owner*—Prescott Lawrence, 196 Madison Avenue, New York.

Foaled 1878. Height 15 hands. Black brown; star; near fore and both hind ankles white.

*Breeder*—Robert Worsley, Suffield Hall, Norfolk, England.

*Sire*—Confidence (158), by Prickwillow (614), by Fireaway Prickwillow (229), by Prickwillow (607), by Norfolk Phenomenon (522), by Norfolk Cob (475), by Fireaway (208), by Fireaway (203), by Fireaway (201), by Driver (187), by Original Shales (699).

*Dam*—An exceedingly well bred bay Hackney Mare. The breeding of this mare cannot now be determined in consequence of the death of Mr. John Grout, the former owner of Fashion.

PLATE II.

sleek, round, trappy thoroughbred with a docked tail; and fake dealers have furnished the market with high-actioned, fat beasts of more or less breeding, docked their tails and by the judicious use of the word Hackney, have found a readier market for horses of unknown breeding and variable value.

The Hackney, on the contrary, is an animal of a fixed family, developed by long and careful breeding (long before its present generic name was applied to it), for a definite object and use from which it derives its name. Because a careless buyer has been imposed upon, or a money-making seller or hasty breeder has furnished impure-bred animals whose progeny revert to the sinister cross and furnish an anomalous individual, is no reason for condemning the whole breed, and even in the importations of the fixed races of the Shire, the Clydesdale and the Percheron, and in the better known families of the Cleveland Bay and the French Coacher, are found examples of mongrel animals from the same cause.

The British Isles had two indigenous races of the horse, the *Equus Caballus Brittannicus*, of which the purest type found to-day is the Shire, and the *Equus Caballus Hibernicus*, of which pure types are still found in Irish horses, but the purest is exemplified by the French Breton and almost caricatured by the Shetland and some Welsh ponies. The oriental horse and his direct descendant, the thoroughbred, were introduced into England at such an early period that it may be added to the two indigenous races, which gives three primitive types of the horse as foundation stock or races, from which by various crosses and selection of the fittest for the object and purposes desired, the several distinctive families of the English horse have been derived.*

* Exclude from these the Clydesdale, which is descended from Dutch horses introduced by the Dutch King on his ascension to the throne of England.

---

PLATE II.—Coaching type.

18 A H.S.B. MATCHLESS OF LONDESBOROUGH. 1517 E.H.S B.
Imp. 1888.

*Owner*—W. S. Webb, Shelburne Farms, Chittenden Co., Vt.

Foaled 1884. Height 15.3 hands. Dark-Chestnut; star; snip; four white ankles.

*Breeder*—N. S. Brough, Londesborough Wold, Yorkshire, England.

*Sire*—Danegelt (174), by Denmark (177), by Sir Charles (768), by Performer (550), by Phenomenon (573). by Wildfire (864).

*Dam*—(463) Lady Lyons, by Lord Lyons (419), by King Charley (392), by Charley (129); *2d dam*—Flora, by Sir Charles (768), by Performer (550), by Phenomenon (573).

PLATE III.

In the advance of civilization, as the industries and commerce required more rapid communication and transit for men and their chattels, and as increasing wealth allowed of the luxuries of sports, which for the better classes replaced the sturdier pleasures of individual combats in warfare and the stout horses required to carry men in armor, the demands of the country were for horses of greater speed and quality. The old Shire was too heavy and slow for post-chaise and coaches and not fleet nor nimble enough for the walls and hedges of the hunting field, nor to win the stakes of the race track. The introduction of the Irish horse, with his closed joints and greater activity, served the purpose at first, but the denser bone and greater heart of the thoroughbred had to be drawn on for greater speed and bottom. In the study of the Hackney two great localities of breeding are represented by the counties of York and Norfolk, and they each furnish distinctive and more or less separate types of the same family. A reference to the human inhabitants of each furnishes us a key to the difference in their selection of the type of horse they bred and had use for. The more thickly-settled county of Norfolk, with its packs of harriers and easy access from London, brought down the friends of John Joricks, who only sported the green at occasional intervals and the pink more rarely, and they asked for more showy action and trappy going, which is furnished by the Irish blood. The great landowners of Yorkshire, with longer distances to travel both by coach and after the fox, demanded more rangy action and a greater stride, and they obtained the lines for these in their horses by additional crossing of the thoroughbred adapted to their purposes. The distinctive type of Yorkshire is seen in the Cleveland Bay, but their Hackneys, while of essentially the same breeding as the Norfolk Hackney, show more of the coaching lines.

---

PLATE III.—Irish type.

14 A.H.S.B. LITTLE WONDER. 2145 E.H.S.B. Imp. 1882.

*Owner*—A. J. Cassatt, Chesterbrook Farm, Berwyn, Chester Co., Pa.

Foaled 1879. Height 14 hands. Bay.

*Breeder*—Frank Beldam, Witchford, Ely, Cambridgeshire, England.

*Sire*—Reality (665), by Confidence (158), by Prickwillow (614), by Fireaway Prickwillow (229), by Prickwillow (607), by Norfolk Phenomenon (522), by Norfolk Cob (475).

*Dam*—by Trotaway (833), by Fireaway (239), by Fireaway (226), by Shales (726), by Norfolk Cob (475). See pedigree extended, Fireaway 242, p. 261. Trotaway's *dam*—Case's Bay Mare, by Fireaway (211), by Adonis (10); *g. dam*—Miller's Brown Mare, by Shales (704), by Marshland Shales (435); *g. g. dam*—by Shales (703), by Marshland Shales (435).

PLATE IV.

The development of the Hackney as a race or family of horses, has been therefore the result of a variable cross of large English horses (mostly Shires), Irish horses and the thoroughbred, and a selection of individuals who have furnished the conformation and quality which has fitted them for the service intended. In order to obtain the high shoulder (so-called knee) action, and stifle (so-called hock) action, the conformation and angles of shoulder and arm, and of the croup and thigh of the Irish horse, have been needed. To obtain the quality and gameness for style and endurance, the blood of the oriental horse has been required.

The vast majority of breeders, dealers and experienced horsemen have an acute perception of the conformation of the animals they handle and recognize that a certain conformation will usually give a certain action, speed, or power of draught or weight carrying, in a given animal, and that the texture of the soft tissues furnishes evidence of the quality, which may be expected in it ; but few have interested themselves in the more fundamental principles which guide the reproduction of these elements. In selection of the fittest, for breeding, in the development of a new family of animals, they have looked rather to the individual and its immediate ancestors and have judged of the results by the rapidly evolutionized change of the soft tissues, rather than to the more remote ancestors, by comparing with which the prominence of the race type is recognized. As the ethnologist recognizes under the dark skin and spare figure of a Hindoo priest and the fair complexion and embonpoint of a New York belle the identity of the Aryan race, by the formation of the skull and bony skeleton, so the scientist can prove in a Corsican pony and the thoroughbred Ormonde an identity of origin. The great Hereford and Shorthorn steers compete in the market as beef animals, and the Friesian (*American Holstein*) cow and her neighbor from the meadows of Normandie are rivals in the dairy, and to the layman the beef animals are

---

PLATE IV —Irish type.

3 A.H.S.B. LADY ALICE. 1605 E.H.S.B. Imp. 1890.

*Owner*—W. S. Webb, Shelburne Farms, Chittenden Co., Vt.

Foaled 1886. Height 15 hands. Chestnut.

*Breeder*—Aaron Beal, Sledmere, Yorkshire, England.

*Sire*—Fimber Fireaway (1482), by Performer (565), by Phenomenon (582), by Fireaway (249).

*Dam*—Bonny, by Skerne Merrylegs (2272), by All Fours 15, by Prickwillow 624, by Performer 550.

PLATE V.

most similar and the dairy animals are like two peas; but the scientist identifies the Durham and the Dutch as of one race, as are the Hereford and the Norman.

The judges of Hackneys, whether they be breeders or umpires in the show ring, have based their decisions principally upon that part of the body which is subject to the more rapid alteration and evolution under the effects of nutrition (feeding, locality and climate) and of functional gymnastics (training and adaptation to special uses). These judges have been experts; they have chosen the best of deep chests which protect the heart, lungs and digestive organs, so essential to strength and endurance; they have recognized the angles and relations of the parts of the propelling members which are required for speed and high and graceful action; they have been cognizant that fine skin, intelligent eye and all that goes to make "quality" is but the expression of the nerve-energy, which alone can obtain great power from the most perfectly-constructed animal machine—in all this their judgment has been correct, and has been accepted and followed by the masses until the desired animal has been approximately obtained. The point of essential interest to the scientist, however, and the one factor which shows at once that the Hackney is a manufactured family and not a type race, is in the variation of the fixed and slowest evolutionized parts of the body, the skull, and pelvis, including the attachment of the tail (*sacrum* and *coccyx*).

A line of Hackney stallions trotted down a show ring, "acting" with the rhythm and style of a drum major's stride, or a row of Hackney mares "camped" for inspection, exhibit as distinctive characteristics of utility for their special service as would be shown by an equal number of Shires, Clydesdales, French Coachers or standard-bred trotters and often more uniformity of general conformation than the latter or a lot of thoroughbreds would show; but an anatomical analysis will show many divergencies of race type. Here will be the broad forehead, straight face and large nostrils of the thor-

---

PLATE V.—Thoroughbred type.

87 A.H.S.B. BELLE 2d. 403 E.H.S.B. Imp. 1891.

*Owner*—George Green, Forest View Farm, Katonah, N. Y.

Foaled 1882. Height 15.2½ hands. Bay; star; snip; three white fetlocks.

*Breeder*—Thos. Nicholson, The Grange, Watton, Hull, England.
*Sire*—Denmark (177), by Sir Charles (768).
*Dam*—(20) Belle, by Fireaway (249); *2d dam*—Violet, by Screveton.

oughbred, with its long horizontal pelvis and coccyx; there will stand an animal with the convex line of frontal and nasal bones, sunken orbital arches and narrow nostrils, and at its other end its Shire blood will be as characteristic in the *short*, oblique croup and depressed sacrum; between and beyond these the widely-set eyes on either side of a flat forehead, the saucy dished nose and the jaunty tail cocked on a *long*, oblique croup, will ornament a beast whose every look and action asks for a good weight on his back or a smart harness and trap behind him to carry his master twelve miles or more in an hour with as much speed and pleasure as the Irish owner of his ancestor ever went to see a fight, evade a tax collector or court his sweetheart.

After the line of the Hackney stallions and mares have been inspected and trotted until the failings of conformation in one have been weighed and balanced against the faults of action in another, and the more deficient have been cast to one side and the ribbons finally awarded, the majority of the winners will be found to show the Irish type. While the judges in this class of horses base their estimation of the merits of the animals upon the general conformation, and take greatly into consideration the angle of the shoulder and the cast of the thigh, their especial confirmation of their opinion is determined by the "action" or manner of going. But little attention has been paid to the head except that it shows intelligence and quality, and the "set" of the tail has been considered more as a question of appearance than of type.

It is evident to a mechanic that closed angles in the articulations of the shoulder (scapulo-humeral) and hip (coxo-femoral) favor the production of the high, quick action of the legs below, which becomes more evident to the eye at the distal extremity, and is so named "knee" and "hock" action. A study of pure types of the three races of horse under consideration shows that in the thoroughbred these angles are open, with the point of leverage (the shoulder blade and pelvis), long and horizontal, favoring speed, as in the greyhound; in the Shire they are closed, with the point of leverage short and tending more to the perpendicular, which favors strength, as in the ox; in the Irish horse, while the angles are well closed and favor quick, high action, the points of leverage are longer than in the Shire and admit of greater speed. It must be still further evident that such a conformation which gives the desired action to perfection, will be more generally found in an animal which has the essential fixed race type, which is found in the head and croup, and that these should be more rigidly considered in judging the Hackney.

That the race characteristic has not been considered to any great extent, and that the Irish horse as a factor in the origin has been to a great extent ignored, except by John Lawrence, will be seen in the extracts from the Hackney Stud book which follow. I regret that I have not available a copy of Lawrence at the present writing, and my own notes from his work are not as complete as I would have them to quote in this article. While all of the following may not bear directly upon the Hackney, it does upon other families of the horse and is of sufficient interest to warrant its reproduction.

### THE DISTINCTIVE NAME OF HACKNEY.*

The oldest surviving appellation for the active riding horse in England appears to be that of Nag, derived from the Anglo-Saxon *hnegan*, to neigh. When the Normans became masters they introduced their own more familiar term *Haquenée*, or *Hacquenée*, the French word derived from the Latin *equus*. This name had been fully adopted into the English tongue in the year 1303. Robert Mannynge, more commonly known as Robert de Brunne, from his being a monk at Bourne, in Lincolnshire, who wrote in that year, uses it in the line "Ilk on his hakneye." In "The Vision of Piers Plowman," written about the year 1350, the word is thus used: "Ac hakeneyes hadde thei none, bote hakeneyes to hyre." Chaucer, who, it is believed, lived for a time in Norfolk, spells the word in two forms—as hakeney and hacknay. *All these old writers used it in the sense of a riding horse for general purposes as distinct from the war horse.*† Both Nag and Hackney continue to be used as synonymous terms to this day. Possibly, the name Trotter, descriptive of the characteristic gait of such a horse, had been used in the vulgar tongue long before we find record of it, for the action is most accurately described by a writer on English customs as early as the year 1170. The earliest use of the word is, however, found in one of the celebrated Paston letters. Margaret Paston, writing to her husband, Sir John Paston, somewhere about the year 1465, from their home "at Heylesdon," near Norwich, has this "*Item:* there he bought for you three horses at Saint Faith's fair, and all be trotters, right fair horses, God save them, and they

* The Hackney Stud book, *Norwich Mercury* office, 1884.

† *Italics* ours.

be well keeped." (St. Faith is a village about three miles from Norwich, which was celebrated for its annual stock fair, until railway communication had made Norfolk buyers comparatively independent of Scotch dealers.) The description of the animals, which had evidently been bought for Sir John's use as road horses, being given without comment, it is evident that there was then a recognized type of horse, in Norfolk at all events, which was commonly known as a Trotter. In the present day we have yet another term, Roadster. Till the close of the last century the description given in advertisements was usually "horses of the road sort," and Roadster does not seem to have been an accepted word till early in the present century. The word itself is not formed in accordance with the established rules which govern the English tongue. Hakluyt, in his "Collection of Travels," published in the year 1600, uses a word *roader*, and we may conclude that it was then in use in Virginia. Roadster, being more easily pronounced, would grow out of such a word as roader. It seems to have been in general use in America, as the common designation for a horse for the road, when as yet it was unknown in England, where the earliest use I have found is in Lawrence's History. In his "Philosophical and Practical Treatise on Horses," published in 1796, he thus sets forth—Vol. 1, pages 267, 268, 3d Edition, 1810—the appellatives:

Horses, for the different purposes of the saddle, were in former days termed Nags, Amblers, Pacers, Stirrers, Trotting-Horses, Hobbies, Great-Horses or Horses for the Buff-Saddle (for war) Hunting-Horses, Coursers, Race-Horses.

The appellatives, whether synonymous or distinctive, in present equestrian use among us are Road-Horses, Riding-Horses, Saddle-Horses, Nags, Chapmen's Horses, Hacks, Hackneys, Ladies' Horses or Pads, Hunters, Running Horses, Racers, Race-Horses, Gallopers, Welter-Horses, Managed Horses, Chargers, Troop-Horses, Post Hacks or Post Horses, Trotters, Cantering Hacks or Canterers, horses which carry double, Cobs, Galloways, Ponies, and Mountain-Merlins.

But in his History (page 117) he uses the novel word Roadster as synonymous with Hackney:

"Our present varieties of the horse, and their denominations, are as follow:

"The Racer, Race-Horse, or Running-Horse; the Hunter; the Charger; the heavy and light Troop-Horse; the Hack, Hackney, Roadster, Road-Horse, or Chapman's Horse; a cloddy compac

Horse or Gelding of this description is now and then styled a *cobb*. The Lady's Horse or Pad ; the Coach Horse, Chariot, and Curricle Horse ; Gig-Horse or Chaise-Horse ; the Machiner and Post-Hack; the Cart and Dray-Horse, Galloways, Ponies."

This last extract shows that the designation Cob is also a modern term.

While new terms have been thus introduced, some old ones have fallen into comparative disuse. Galloway was a general name for the "best nags" when Duke of Newcastle wrote (A. D. 1660). Hobby was the general title given to the Irish Horse, by Thomas Blundeville, of Newton Flotman, near Norwich, who wrote in the year 1558, "for the most part they be amblers, and therefore very meet for the saddle and to travel by the way," and the name was in common use in England till the close of the last century. The old sayings, "Every man has his Hobby," and "He rides his Hobby," are familiar. Lawrence says the principality of Wales formerly bred scarcely any other breed than hobbies, which the Duke of Newcastle characterizes as "excellent good" nags. The Cornish "good nags" of the Duke of Newcastle's day were correctly spoken of by Lawrence as "gonellies," Goonhilly Down on the Lizard Peninsula, being the home of the small, strong pony which was almost the only variety of horse in use in Cornwall, as a pack-horse, before roads became common. Foresters was the name given to the small nags which, till the middle of the 18th century, were bred and sent out in great numbers from the New Forest. While the three last named terms and others mentioned by Lawrence, have fallen into entire disuse, Galloway and Pony have been adopted as convenient general terms to describe height ; Cob has been generally accepted to signify a horse below 14 hands 3 inches, and which is also compact, low and able to carry a weight out of proportion to its height ; and the general name applicable to horses for the road, as saddle or driving horses, which range in height above fourteen hands, continues to be Hackney or Roadster.*

The Rev. Samuel Pagge, a noted Norfolk antiquary, in his Essay on Coaches, to be found in his "Curialia Miscellanea," (A. D. 1817,) page 280, says :

"The French word Haquenee implies a common horse for all

* The term Cob is being very generally used in America by some dealers and a certain class of people to designate any stout, compact built horse, without reference to height, and even by others, for animals of 15 hands or more, either as a noun or in the adjective form "cobby."

purposes of riding, whether for private use or for hire ; generally an ambler as distinguished from the horses of superior orders, as the palfrey and the great horse. The former of these are often called pad nags, and were likewise amblers, while horses for draught were called trotting horses (Northumberland Household Book, p. 127), so that tne Haquenee was in fact, and in his use, distinct from all the rest and inferior in rank and quality."

The difference in meaning between the words Hack and Hackney—save that we yet speak of a Park-hack and a Lady's-hack—which has been established by customary use, is well stated in the following extracts from William Taplin's "Sporting Dictionary and Rural Repository of General Information" upon every subject appertaining to the Sports of the Field, published in the year 1803 :—

"HACK.—Any horse appropriated to every kind of purpose (and upon which no great estimation or value is placed) it has been the custom from time immemorial to distinguish by the appellation of Hack. Custom, however, has permitted a slight deviation from a practice of long standing, and a Hack is now generally understood to imply the idea of a hired horse.

"HACKNEY, in the general acceptation of the word with the sporting world, is a horse superior to all others upon the score of utility ; being rendered subservient to every office of exertion, speed, or perseverance, or in other words, to all the *drudgery and labor* of his situation, from which his contemporaries, the racer, the hunter, and the charger, by the *imaginary* superiority of their qualifications and pampered appearance are always exempt. It is the peculiar province of the Hackney to carry his master 12 or 15 miles in an hour to covert (where the Hunter is in waiting), and sometimes to bring back the groom with greater expedition, whose engagements may probably have occasioned him to be much more in haste than his master. It is in the department of the Hackney to encounter and overcome emergencies and difficulties of every description ; his constitution should be excellent and his spirit invincible ; he must be enabled to go five and twenty or thirty miles at a stage, without drawing bit, and without the least respect to the depth of the roads, or the dreary state of the weather ; and if he is not equal to any weight in these trying exertions he will be held in no estimation as a Hackney of Fashion."

The Romans would appear to have distinguished accurately no fewer than eight classes, according to the action of the horse or the use to which it was applied ; the hackney, or common road horse, being denominated *Itinerarii*, and the amblers or pacers as *Ambulaturarii*. We may assume that these several kinds of horses were in general use in Britain, seeing that the Roman settlers were men of wealth and lovers of comfort, as is proven by the recently discovered remains of villas, no less than by the magnificent workmanship of their roads and camps yet existing. Naturally they would have improved the possibly indigenous horse stock which the British had in use when Julius Cæsar landed, and which he admits were so highly trained as to have given the islanders a temporary advantage over the invading Roman foot soldiers. When Rome was obliged to withdraw her army and Britain fell a prey to the Saxons, the horses so improved would necessarily have been left in the Island, and, doubtless, served the purpose of the new settlers.* The Danes who were the next invading people must certainly have helped to maintain the excellence of the horse stock. We know that in the home of the Norsefolk there was a breed of horses, small-sized generally, and always low, while Lawrence suggests that the duns and sorrels or chestnuts of Norfolk or Suffolk owe the color to a cross from the Norwegian by the use thereon of Norfolk stallions.† It is almost beyond dispute that the district owes to the Norsefolk its polled or hornless cattle, and there is no little probability that to the influence of the same people, selecting from British stock, and crossing with their own esteemed little horses, we owe the variety of road horses for which Norfolk and its borders, and Yorkshire, have been long famous. The horses of Norway, we are told, are "round-made, but with clean heads and limbs ; their best pace is the trot, which, indeed, is the characteristic pace of the Northern, as the gallop is of the Southern, horse. They are so sure-footed in their own rough country as to be equal to mules in that rare quality." (Lawrence's History, p. 55 ) The fact that the trotting horse was, in the last century, found most plentifully in those districts of the kingdom where Danish settlers had left indelible marks of occupation and habitation, warrants the assumption that to Norse horse stock we in great measure owe this characteristic action.‡

---

*History shows, however, that the Romans were importers of horses, as were the Italians of Mediæval times, and the only horse imported into England to any great extent has been the Oriental horse.—H.

†Color is not accepted by scientists as any indication of race in the domestic animals.—H.

‡The Danish horse had a Roman nose and a roach back, as seen in the black hearse horses in Paris to-day, and in the Luxembourg coach horses.—H.

### Improving the Breed of Road Horses by Legislation.*

In the year 1495 we have the Act 11th, Henry VII, c. 13, the first attempt of the Legislature to increase and improve the English breed of horses. We may suppose that the Wars of the Roses, which greatly reduced the population of England, and caused, much land to go out of cultivation, were the true cause for the neglect of horse breeding and for the degeneracy which the preamble to the Act admits, though an altogether different reason is assigned.

In the year 1530-31, there is a further Act, 22d, Henry VIII, c. 7, strengthening the Act of 1495 by increasing the penalty to 40s. per "poll" for removing out of the real many "horses, geldynges, mares, coltes, oxen, steres, bullocks, calves, kyne, or shepe," without the King's special license, given under his Great Seal of England.

The next Act, of the year 1540, 32d, Henry VIII, c 13, "For bryde of Horses," seems to have been passed by the House of Lords with great enthusiasm. The entry in the Journals, under date June 15th, 1540, translated, reads thus :—At length the bill is read this day for encouraging the breed of Horses of a larger stature, and dispatch with unanimous consent, and without a dissentient voice.

The only other remaining Act of Henry VIII's reign—which continued until the 21st, James I, 1623-4, and was then repealed, with many other Acts, which we may suppose had fallen into disuse—was that of 33d Henry VIII, c. 5 (A. D. 1542). It is of great interest, because it shows how greatly trotting horses had become to be valued.

It therefore provided that "every Archebusshoppe and Duke of this realme" shall "have, fynde, kepe, susteyne, and meyntene . . . seven-stoned trotting horses for the saddle, every horse of the same to be in age three years and upwarde, in height xiiij handfulles;" marquises, earls, and wealthy bishops to keep five such horses; other bishops, viscounts, and wealthy barons three such horses; less wealthy persons of similar rank to keep two such horses; while "all and every person temporall, not afore mencyoned, whose Wiff after the feast of Saint Michaell tharchaungell, next hereafter mentioned in this Acte, shall were any gown of sylke, or whos Wiff shall were any Frenche hood or

*English Hackney Stud Book, page 9.

bonnett of Velvett, with any habiliment past or egge of gold, perle, or stone, or any chayne of gold about their nekke, or in their partlette, or in any apparell of their bodie shall . . . have, fynde, kepe, susteyne, and mayntene, as is aforesaid, one-stoned trotting horse for the saddill."

This singular old Act was drawn so precisely that it would seem impossible to escape its penalties, a proviso being made to meet even such a case as a lady wearing velvet in the lining or any part of the gown other than the cuffs or in her kyrtell ; or her wearing any petticoat of silke; her husband being, thereupon, obliged to find and maintain one-stoned trotting horse. The last clause is, however, the most valuable, because it plainly distinguishes the trotting horse of that day from the cart horse, and the Sumpter horse, from one of which varieties some modern authorities have supposed the English Hackney or Roadster to have been descended. The clause reads as follows :

"Provyded also that Cart Horses or Sumpter Horses shall not be takyn, reputed, or reckned for any suche horses whiche any person is or shalbe bounden to kepe by vertue of this Acte."

The year 1558, which saw the accession of Elizabeth to the English throne, is noteworthy also for the production of the first English book on horses : "The foure Chiefest Offices belonging to Horsemanship . . . which books are not only painfullie collected out of a number of authors, but also orderly disposed and applied to the use of this our countrey : By Master Blundeville, of Newton Flotman, in Norfolke."

*Blundeville gives an interesting account of the light Irish horse, which shows that an excellent race of nag horses was to beo fund in that island three hundred years ago (p. 7, ed. 1609) :

"The Irish Hobbies is a prettie fine horse, having a good head and bodie, indifferently well proportioned, saving that many of them be slender and pin-buttocked; they be tender mouthed, nimble, light, pleasant, and apt to be taught, and for the most part they be Amblers, and therefore very meet for the saddle and to travele by the way ; yea and the Irish men bothe with darts and with light spears to use to skirmish with them in the field. and many of them do prove to that use verie well, by means they be so light and swift ; notwithstanding I take them to be very nesh (soft) and tender to keep, and also to be somewhat skittish and fearful,

---

*The Hackney Stud Book, page 19.

partly perhaps by nature and partly for lack of good breaking at the first."

The English breeders of horse-stock Blundeville's day would appear to have been as desirious as they are now of having a wide choice. He says:

"Some man would perchance have a breed of great trotting horses meete for the warre and to serve in the field. Some others againe would have a breed af ambling horses of a meane stature, for the journey and to travel by the way. Some, perhaps, would have againe a race of swift runners to runne for wagers, or to gallop the Bucke, or to serve for such like exercises of pleasure. But the plaine countryman would perchance have a breed only for draught or burthen."

The "plaine countryman" would be such yeomen as were Blundeville's neighbors in and about Norwich and in the county of Norfolk. At that time these squires and yeomen had their own favorite breed, which, as will be shown presently, could trot, plow, or hunt, while they were also excellent for the draught—a breed now represented only by the Norfolk Hackney horse.

*Mention is also made of the "light horsemen here in England" riding "in the warres," either on ambling colts or on trotting colts, though the latter were kept "partely for their servantes to ride on, and to carrie their males and cloke-bagges after them." That these English horses were stout and able to stay is evident from Blundeville's assertion that our men "will not stick to ride forty, fifty, three-score mile a day."

In Blundeville's days the best colors for horses were held to be "the browne bay, the dapple gray, the bright bay, the rone, the white liard, the pure black with a white star in his fore head."

At about the commencement of the 17th century are found references to the value of the oriental horse, and we find accounts of the introduction of the Barb, Turkish and Asiatic horses.

Michel Baret, of Lincolnshire, 1618, says:

"Certainly English horses are the best horses in the whole world for all uses whatsoever, from the cart to the mannage, and some are as beautiful horses as can be anywhere, for they are bred out of the horses of all nations. . . . Do not think to buy delicate-shaped horses like the Spanish horse, Barb or Turk, but they are handsomer horses than commonly Dutch horses are. . . . In the west country my Lord Paulett's ancestors had a

* English Hackney Stud Book, page 22.

good breed of horses, and by chance now and then my Lord of Pembroke did breed, but I never heard of any rare horses of his race. . . . In Cornwall there are good naggs, and in Wales excellent good ones, but in Scotland the Galloways are the best naggs of them all. . . .

"For English mares there are none like them in the world to breed on, but then you must chuse them fit for such horses as you would breed."

About the commencement of the 18th century the systematic breeding for the improvement of the local race was begun, and the Arabian was called on to increase size as well as quality, as is seen in the following :

* In Cosgrove's *Norwich Gazette,* of the year 1725, is seen the advertisement of an "old gray padd stallion" in use in the county of Norfolk : his height 14 hands. (Advertisements in the *Norwich Mercury* of the year 1727 fix the height of a large, stout coach gelding at 15 hands. A bay Hackney is advertised in the following June as lost—his height, too, is 15 hands. Other horses are advertised in July, 1778, as from 13 to 14 hands.) Then there is the first sign of the improvement which doubtless had gone on from Blundeville's time. On April 12th, 1729, there is an advertisement in the *Norwich Mercury* which reads as follows: "Lately come into Norfolk, a famous stallion, an Arabian, by the size 15 hands 3 inches, and strength proportioned." The next mention of an introduction into the county is found in an advertisement in the same journal dated May 2d, 1741. It speaks of a "very large bay stallion, exceeding strong ;" height 15.3 hands; its sire, "a fine, strong hunter of Squire Pulteney's near Beverly, Yorkshire, and was bred by a mare of Farmer Bell's, upon York Wolds : the same mare bred the noted running mare called Lady Leggs, and afterwards Painted Lady."

† John Lawrence in his "Treatise," published in 1796, thus speaks of the results :

"No importation of saddle horses has ever taken place within the present century. . . . The original breed of English horses has been long since entirely extinguished by that general improvement which has pervaded every quarter of the country. A curious observer may, nevertheless, form a very good estimate of

* English Hackney Stud Book, page 34.

† Ibid, page 35.

its figure and merits by examining our common road hacks, which show little or no mixture of foreign blood, and the lower kind of farmers' horses, to the breed of which little or no attention has been paid." (Vol. I, pp. 92-3.)

* Improvement was noteworthy in some of the Welsh counties, especially in Caermarthen, where blood stallions were brought every season from the English counties. Welsh horses, in fact, in the course of a few years were commonly known as Mountain Merlins, because a thoroughbred of that name had produced such marked improvements in the stock. The report from Montgomeryshire says :

"There are many saddle horses reared of all descriptions, from the hunter to the Welsh pony, but by far a greater number of the latter; indeed, I believe that Montgomeryshire breeds more of those ponies than any two counties in North Wales."

Devon had its own characteristic variety of Cobs, which by the use of thoroughbred blood and judicious selection, furnished that county with fairly good road horses Cornwall, as we have already mentioned, had its Goonhillies and its Gossmoor ponies—small, hardy stock, well adapted in a hilly country, for the pack saddle, carts not being yet used in that country.

† Fortunately Mr. Cooper, R. A., has preserved for us (and the picture is reproduced in the *Sporting Magazine* of August, 1820) a faithful portrait of this sort of trotting cart horse, and is said to be inserted "as a specimen of that useful kind of horse which is equally adapted to draught or the saddle, and which is suitable to many purposes and situations." This trotting horse is a genuine Norfolk Cob, with plenty of hair, short legs, a good lengthy barrel and only wanting a little blood to improve his quality. Lawrence fully bears out Marshall in his assertion of the merits of this trotting Norfolk and Lincolnshire horse.

‡ The following communication from Mr. John Armstrong Storey, written in November, 1878, from 78 Shaftesbury Road, Hammersmith, is of interest as being the experience of a breeder of Hackneys, who himself knew some of the most noteworthy horses in Norfolk :

"I learn with much pleasure that you have engaged to compile a stud book of Norfolk Hackneys. As a breeder of that class of

*The Hackney Stud Book, page 44.

†Ibid, page 47.

‡Ibid, page 74.

horse, during 40 years of my residence in Norfolk, I trust a few remarks from my pen will not be deemed presumptuous. The first point for consideration appears to be, 'What is a Hackney?' Doubtless it is the produce of *thoroughbred and old Norfolk strains** of blood so blended and cultivated that an almost distinct race is established, combining all the desired characteristics of the two families in an improved model frame. The task is by no means an easy one, and may take years of study to effect, for continual disappointments will arise through a tendency to breeding back to one or other of the original stocks. Under the most favorable circumstances it must be a tedious process, as no horse can pass muster as a Hackney unless he can boast of three generations untainted by nearer proximity to either of the original roots of parentage. This rule, which has been accepted through all ages, I hold to be imperative, as the safest and soundest definition of the Hackney strain of blood. It is necessary in blending to study closely the symmetrical points required. Perhaps they can best be defined as exhibiting the elegance of the thoroughbred above the line, with the substance of the cart horse beneath that limit of demarcation; but the shoulder must be deep and lie well back, with withers well upraised, the arm long and full of muscle, the leg-bone short, flat, and largely supported with sinew, the fetlock short and strong, the foot circular and tending to be upright, the frog well hollowed out and pliable, the thigh must be muscle itself, hock clean and accurately jointed, the hind rib being short, that he may be better "Gang away, and tuck his haunches in." Sidney, in his "Treatise on the Horse," dates back the origin of Norfolk Hackneys to Bond's Norfolk Phenomenon (522). This proposition is too absurd to admit of a moment's consideration. What kind of horse does he suppose carried our forefathers, with their wives on pillions behind them, over the almost untracked heaths and commons of Norfolk, during the 18th century? Surely not the cart horse; certainly not the thoroughbred – a burly yeoman, of say 16 stone, with Amphillis, of 9 or 10 stone, on pillion behind, would have ridden through the thoroughbred's delicate form in a very short period of time, so far as my memory serves."

†M. de Thannberg, who for nearly forty years was connected with the Government Studs of France, and who is considered the highest authority in that country on all subjects connected with horse-breeding, wrote:

* Italics ours.

† English Hackney Stud Book, page 82.

Allow me to say a few words on your Norfolk trotters, or roadsters. I place the greatest reliance on this breed; for it has been the source of the great amelioration in our horses, which has been produced in France.* Although these horses are not very high bred, they are of very similar conformation. They invariably transmit to their offspring all their qualities, their action, their courage—in one word, all that constitutes the requisites for a good troop horse. Unfortunately for England, Norfolk stallions are becoming very scarce; foreigners pick them up wherever they find them, and the want is felt sensibly.

Mr. E. Greene, M. P., says: one was very likely to get a horse fit for harness purposes, by a well-bred Norfolk stallion, than by a thoroughbred, unless the latter had high action, which was not suitable for racing. He would have a trotting stallion, with all the breed that could be possibly got, and would not recommend a heavy trotting stallion. He mentioned Ambition (C. Bearts') as the kind of horse which got foals with great action and good appearance, which he thought most valuable for harness purposes. Mr. Green concluded his evidence thus: "I only hope that I am not misunderstood in my advocacy of using a trotting stallion. I do not despise a thoroughbred horse, because I believe the thoroughbred horse is the foundation; but what I say is this, and in our present emergency particularly, we want this trotting horse, as being adapted to the mares that are left, and likely to get good action, and that action is valuable in the market, and is why I recommend the breeding from them. But I wish to be quite understood that the thoroughbred horse is to be the foundation; and if I could get a thoroughbred trotting stallion I should breed from him and nothing else, if I could get his action high enough."

The Earl of Charlemont said he found the breeding of harness horses on his Irish estate paid better than any other—harness horses for all purposes. Most of the half-bred mares were put to a Norfolk horse, whose action was very superior and his temper perfect. The farmers in the neighborhood took kindly to this horse. His experience was that not so much quality was wanted in the stallion as in the mares for that class of horse. He did not see the horse till he had seen his stock, and bought the horse with-

* The Norfolk trotters were purchased by the French Government to breed on the Breton mares. This was not considered a cross, as one of the gentlemen who selected the horses, M. Trasbot, was then satisfied of the identity of the Irish blood, and its progeny the Norfolk, with the Breton.—*Personal communication to myself.*—*H.*

out having seen him. Two of the best horses in London for the last three years were got by this horse.

There was in the Hackneys of that day a union of substance and action which we do not so often witness at present, and which is perhaps now more readily to be met with in the hack and hunters of Ireland—considerable annual importations of which have of late years occurred.

---

The foregoing abstracts from the English Hackney Stud Book are not as lengthy as I would have made them if space would permit, but they serve to show the class of horse and its locality of origin, which furnished the mares for the foundation of the Hackney. The thoroughbred was then introduced and used extensively to improve the speed and the quality of the native breeds, but the thoroughbred was selected when he showed trotting qualities and suited the mares, and *while he has considerably modified the conformation of the old-fashioned Hackney he has not destroyed its type.*

*En resumé*, the Hackney is a family of the horse which has been developed by careful selection in crossing and metissage from three races,

The Shire—*Equus Caballus Britannicus*,
The Irish Horse—*Equus Caballus Hibernicus*,
The Thoroughbred *Equus Caballus Asiaticus*, until it transmits to its progeny its characteristics with almost the fixity of transmission found in a pure race.

The essential characteristics desired are:

From the Irish Horse—

Type of head and croup.
The angles of the articulations and their result—high-styled action.

From the Thoroughbred—

Density of bone,
Nerve energy,
Quality.

From the Shire—

Elimination of all trace, except perhaps a tendency to size. (By atavism, however, the Shire type is sometimes most unmistakable.)

155 West 56th Street, New York.

## Figures and Tables

**PLATE I. f1:**
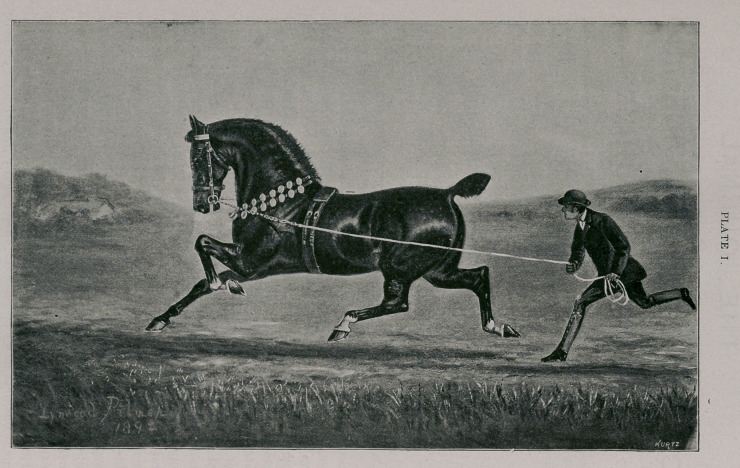


**PLATE II. f2:**
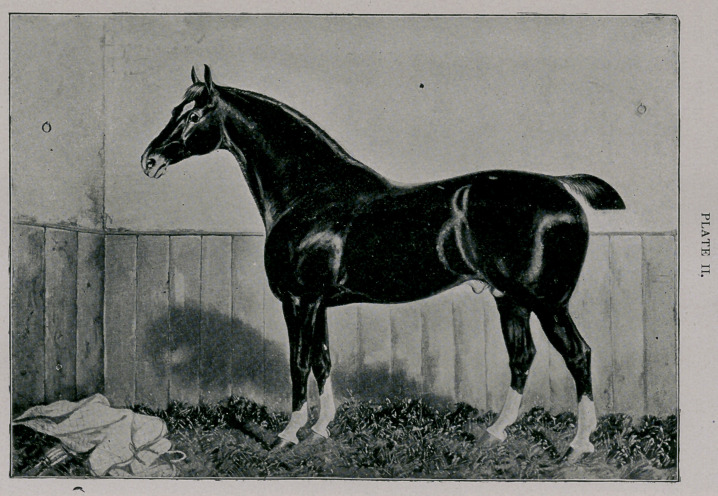


**PLATE III. f3:**
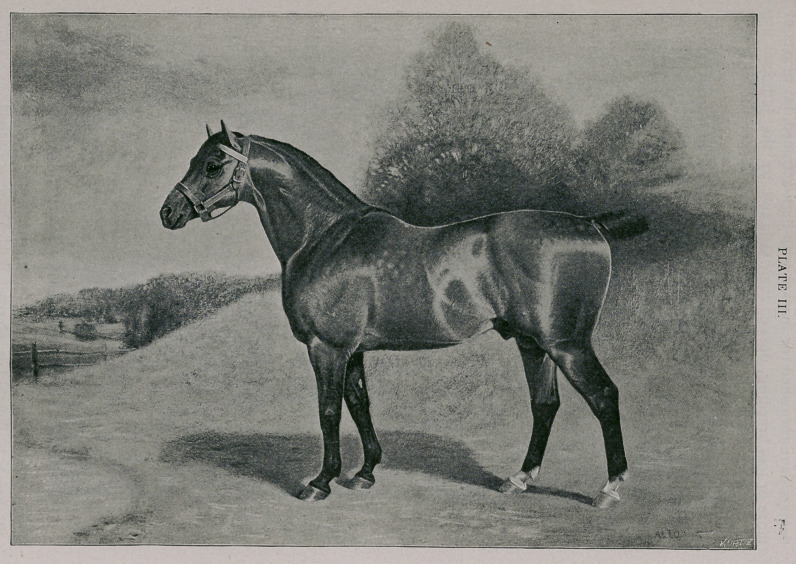


**PLATE IV. f4:**
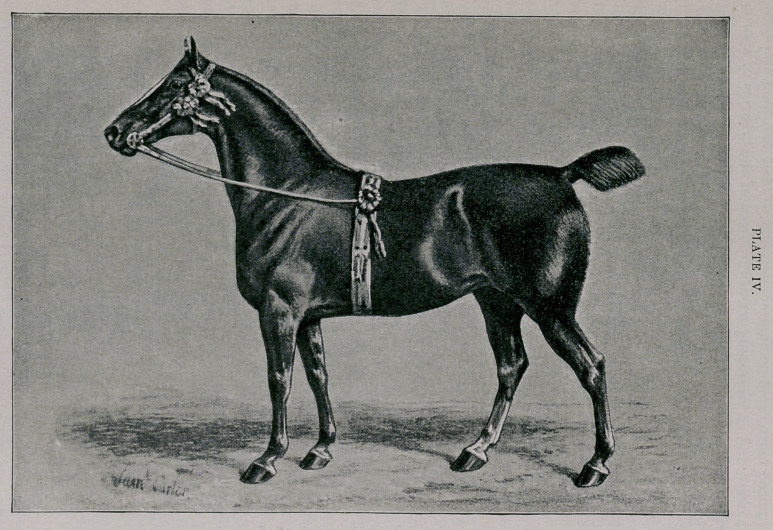


**PLATE V. f5:**